# Benefits Cliffs and Financial Stability for the Direct Care Workforce

**DOI:** 10.1093/geroni/igaf122.1442

**Published:** 2025-12-31

**Authors:** Sarah Angell, Susan Chapman, Jiyeon Kim, Stephen McCall

**Affiliations:** PHI, New York, New York, United States; University of California, San Francisco, San Francisco, California, United States; PHI, New York, New York, United States; PHI, New York, New York, United States

## Abstract

Our research examined the risk of benefits cliffs among direct care workers (DCWs) in Florida, Kansas, Massachusetts, New Mexico, and Virginia by analyzing effective marginal tax rates (EMTRs), which measure how much of a worker’s additional earnings are lost due to higher taxes and reduced public benefits. A benefits cliff occurs when the EMTR exceeds 100%, meaning workers end up financially worse off despite earning more. Using the American Community Survey (ACS) to identify DCWs, we estimated earnings thresholds for benefits cliffs with the Federal Reserve Bank of Atlanta’s Policy Rules Database (PRD), which calculates benefits and taxes based on family characteristics. We modeled three consecutive $5,000 wage increases and calculated overall resources as take-home family income plus benefits. We find that DCWs face the risk of benefits cliffs. In Virginia, 8.8% of workers would encounter a cliff after a first $5,000 raise, 6.4% after a second, and 5.3% after a third, with 16.3% experiencing at least one cliff across raises. Median EMTRs range from 35.4% to 36.2% across raises. These cliffs and high EMTRs can leave DCWs with few financial resources even after wage increases. Given low wages, unstable work hours, and high poverty rates, policies to improve economic stability for DCWs are critical. To mitigate the effects of benefits cliffs and improve financial stability for DCWs, policymakers can make changes to public benefits programs including adjusting income limits and asset limits, while employers can engage DCWs in strategic discussions about wage increases and scheduling changes.

